# Frontiers in Innovative Materials and Technologies for Oil–Water Separation

**DOI:** 10.3390/polym17121635

**Published:** 2025-06-12

**Authors:** Jikun Jiang, Shunda Wan, Cheng Wen, Li Tang, Ning Xu

**Affiliations:** School of Environmental Science and Engineering, Nanjing Tech University, Nanjing 211816, China; jkj@njtech.edu.cn (J.J.); 202361202073@njtech.edu.cn (S.W.); 202361202068@njtech.edu.cn (C.W.); tangli@njtech.edu.cn (L.T.)

**Keywords:** biomass substrates, membrane separation, multifunctional separation materials, oil-contaminated wastewater, oil–water separation technology, superhydrophobic/superoleophilic materials

## Abstract

Oil-contaminated wastewater represents a major source of industrial pollution, posing significant risks to both the environment and human health. Traditional oil–water separation methods, including gravity separation, centrifugal separation, and air flotation, are limited by their processing efficiency and scope of applicability. In recent years, innovative oil–water separation technologies have gained considerable attention, particularly those utilizing adsorption, filtration, and membrane separation, owing to their high efficiency and environmental sustainability. Separation materials derived from biomass substrates—such as cellulose, chitosan, and lignin—along with metal-based membranes and polymeric filters, have shown remarkable performance. This is especially true for superhydrophobic/superoleophilic and stimuli-responsive materials, which excel in separating complex emulsified oil systems. This paper provides a comprehensive overview of the strengths and limitations of current separation technologies and explores the potential applications of multifunctional materials in treating oil-contaminated wastewater, offering both theoretical insights and practical guidance for advancing green, efficient oil–water separation solutions.

## 1. Introduction

With the continuous advancement of industrialization, the global annual discharge of oil-contaminated wastewater has reached hundreds of billions of tons [1,2,3]. In response, many countries have implemented stringent regulations to limit the maximum concentration of oil in wastewater to levels ranging from 5 to 100 mg/L [4,5], pushing industries to reduce wastewater emissions. However, economic constraints and technological limitations continue to make the treatment of oil-contaminated wastewater both challenging and costly. The water-in-oil and oil-in-water emulsions present in such wastewater are the primary pollutants discharged into aquatic ecosystems. Their release can severely impact drinking water sources, infiltrate groundwater, and cause groundwater contamination. Moreover, these pollutants can spread to the atmosphere and soil, resulting in significant environmental degradation and resource loss while also threatening human living conditions [6]. Additionally, harmful compounds such as phenols, petroleum hydrocarbons, and polycyclic aromatic hydrocarbons in oil-contaminated wastewater can hinder the growth and development of both plants and animals. Therefore, the efficient treatment of oil-contaminated wastewater is crucial for environmental protection and sustainability [7,8,9].

The droplet size is a critical factor in the treatment of oily wastewater, directly influencing both separation efficiency and the selection of treatment technologies. Oil–water mixtures typically exist in three physical forms based on the state of the impurities: immiscible oil–water mixtures, free oil–water emulsions, and emulsions stabilized by emulsifiers. These emulsions can further be classified as either “water-in-oil” or “oil-in-water” types [10].

Immiscible oil–water mixtures are quite common and are characterized by an oil film or layer floating on the water’s surface. The oil droplets in these mixtures are typically larger than 100 μm in diameter, which makes them relatively easy to separate [11]. Free oil–water emulsions, on the other hand, consist of tiny oil droplets, either liquid or solid, suspended in water. These emulsions are unstable and can be influenced by factors such as water temperature, flow conditions, and gravity. As a result, the oil droplets may aggregate into larger particles that float on the water’s surface, with sizes typically ranging from 10 to 100 μm [12].

Oil–water emulsions are classified into oil-in-water (O/W) and water-in-oil (W/O) types, depending on the type of dispersed and continuous phases. In oil-in-water emulsions, oil droplets are dispersed as small liquid spheres within water, with a stable interfacial film formed at the oil–water boundary. This structure allows the oil droplets to maintain excellent dispersion and stability in the aqueous phase. In contrast, water-in-oil emulsions feature water droplets dispersed as tiny liquid spheres within oil. The particle size of emulsified oils in these systems typically ranges from 0.1 to 2 μm, making them difficult to separate using conventional treatment methods. As a result, these emulsions can only be effectively separated through physical or chemical processes, but such methods often prove inefficient at removing small oil droplets and separating emulsified oil [13].

The most critical aspect of treating oil-contaminated wastewater is reducing the oil content to minimize environmental pollution. Optimizing oil–water separation processes to achieve eco-friendly solutions and improve the removal efficiency of oil substances remains a key challenge [14]. This requires treatment methods that are not only cost-effective and efficient but also prevent secondary pollution. In response to these challenges, there has been significant progress in developing innovative technologies and new materials to address the complexities of wastewater treatment.

Materials used for oil–water separation are generally classified into two categories: “superhydrophobic/superoleophilic” (oil-removing type) and “superhydrophilic/superoleophobic” (water-removing type). Superhydrophobic materials have garnered considerable interest from researchers as alternative solutions for oil–water separation due to their distinct interactions with the two liquids. These materials are designed to prevent water permeation while allowing oil and non-polar solvents to pass through [15,16]. The water contact angle (WCA) is commonly used to characterize the hydrophobicity of materials. It measures the angle between the tangent to the liquid–gas interface and the solid surface. If the WCA exceeds 90° but is less than 150°, the material is considered hydrophobic; if it exceeds 150°, it is classified as superhydrophobic. Currently, hydrophobic materials have been shown to be highly efficient in oil–water separation processes [17,18].

This paper provides a comprehensive analysis of the advantages and limitations of current technologies and investigates the potential applications of multifunctional materials in treating oil-contaminated wastewater. It offers both theoretical insights and practical guidance for advancing the development of environmentally friendly and efficient oil–water separation technologies.

## 2. Traditional Oil–Water Separation Methods

Traditional separation methods, such as gravity separation, centrifugation, and air flotation, each operate based on different principles, leading to varying levels of efficiency. Gravity separation is effective for larger oil droplets (greater than 10 μm) but exhibits lower efficiency when dealing with smaller droplets, making it less suitable for fine oil removal. In contrast, centrifugation excels at separating fine oil droplets (less than 10 μm) and can achieve rapid separation. Air flotation, on the other hand, is capable of treating both fine oil droplets and emulsified oil, offering high treatment efficiency across a broad range of oil types. Among the traditional separation techniques, gravity separation, centrifugation, and air flotation are most commonly used.

### 2.1. Gravity Separation Method

Gravity separation is a technique that exploits the differences in relative density between oil, gas, and water to achieve phase separation. Under consistent external conditions, such as pressure and temperature, these three phases maintain a stable equilibrium. According to Stokes’ law, components with lower relative density rise to the upper layer, while those with higher density sink, allowing for effective separation [19]. Gravity settling devices, which are designed based on this principle, are a typical example of gravity separation methods. These devices are characterized by their simple structure, ease of operation, and suitability for large-scale wastewater treatment applications.

Based on practical experience, Hazen proposed the “shallow pond theory,” which suggests that the settling efficiency of dispersed particles, as opposed to flocculated particles, during gravity sedimentation can be evaluated as a function of the settling velocity and the pond’s surface area, independent of the pond’s depth and settling time [20]. However, this method is time-consuming as it requires sufficient time for the mixture to separate. Furthermore, it is not effective for separating stable emulsified liquids, highlighting the need for alternative methods to improve oil removal efficiency. In addition to gravity sedimentation, centrifugation can be used to accelerate the deposition of solid particles, thereby enhancing the separation of oil and water phases.

### 2.2. Centrifugal Separation Method

Centrifugal separation technology is an effective method that leverages the density differences between oil and water. By subjecting oil–water mixtures to high-speed rotation, this technology utilizes varying centrifugal forces to achieve efficient separation. Centrifugal equipment can attain rotation speeds that generate forces several hundred times greater than gravity, enabling the rapid separation of oil and water with relatively compact equipment [21,22]. However, the inclusion of moving components within the equipment adds complexity, making routine maintenance more challenging and hindering its widespread industrial application.

The primary centrifugal separation device is the hydrocyclone, which separates continuous-phase liquids from dispersed-phase solid particles, droplets, or bubbles through physical forces. A key factor influencing separation efficiency is the density difference between the dispersed and continuous phases. The larger the density disparity, the easier the separation process, leading to more effective separation outcomes [23].

Similar to separation in a gravitational field, the particle diameter of the dispersed phase plays a crucial role in separation efficiency when there is a constant density difference between the two phases. Larger particle diameters result in greater velocity differences during the equilibrium state, making it easier to separate the two phases. This principle is also applicable to the centrifugal separation process in hydrocyclones.

### 2.3. Flotation Method

**Figure 1:**
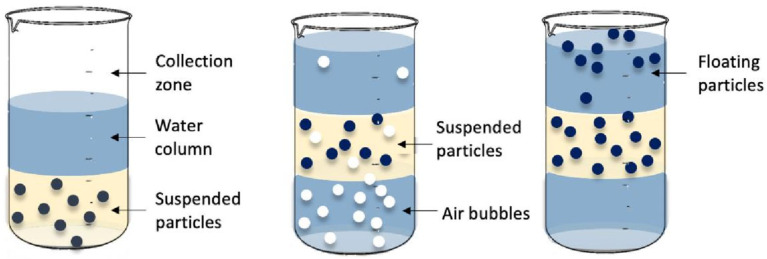
Flotation process: suspended particles rise to the surface for removal, resulting in clarified water at the bottom [13].

The flotation method utilizes highly dispersed microbubbles as carriers to attach to oil contaminants in oily wastewater, aiding their rise to the surface, where they form a froth. The addition of a collector, typically a surfactant that can be recovered at the end of the process, helps to clarify the water at the bottom, thus enabling phase separation, as shown in Figure 1 [24,25,26]. This technique is well established and demonstrates high oil removal efficiency, consistently achieving oil–water separation efficiency of up to 80%, even under high load conditions and short processing times. However, the complete removal of surface scum remains a challenge [27].

The flotation process depends on the surface and interfacial properties of both the bubbles and the encapsulated particulate system. Because the colloidal particles in oily wastewater typically have small diameters, the use of microbubbles is essential for effective separation. However, the number of microbubbles generated per unit volume of water during the flotation process is limited, which is a crucial factor for the separation of suspended solids [13].

Recent studies have shown that the combined effect of nanobubbles and microbubbles can significantly enhance oil–water separation performance and improve the ability to capture pollutants in water. The integration of both microbubbles and nanobubbles in assisted flotation processes demonstrates promising applications in the treatment of both drinking water and wastewater [24,28]. Furthermore, the efficiency of flotation is influenced by various factors, including air resistance, bubble size and distribution, solute concentration, particle size, gravity, surface tension, and the type of surfactant used [29].

In summary, the gravity separation method is known for its simplicity, efficiency, and lack of energy consumption, making it ideal for the preliminary treatment of low-concentration oily wastewater. However, additional measures are necessary for handling high-concentration wastewater. The centrifugal separation method, while effective, faces challenges related to maintenance due to the complexity of its equipment, as well as high investment and energy costs, limiting its use primarily to laboratory settings and confined spaces. The flotation method is highly effective for treating emulsified oil–water mixtures, but its practical application requires careful consideration and adjustment to suit specific conditions.

## 3. Novel Oil–Water Separation Methods

Although traditional oil–water separation methods are relatively simple to operate and have been widely used in the past, they have several limitations, including low efficiency, complex equipment, and high energy consumption. In contrast, innovative oil–water separation techniques have shown significant improvements in efficiency, environmental sustainability, and cost-effectiveness. These advanced methods mainly include electrocoagulation, adsorption, filtration, and membrane separation techniques.

### 3.1. Electrocoagulation

Electrocoagulation (EC) represents an effective electrochemical approach for treating oily wastewater [30]. Under an applied direct current, sacrificial iron or aluminum anodes undergo dissolution, releasing metal ions, while simultaneously generating potent oxidizing agents (e.g., oxygen, hydroxyl radicals, chlorine) and microbubbles at the electrodes. These products act synergistically to destabilize emulsified oil droplets and degrade organic contaminants, thereby facilitating oil–water separation [31,32]. Compared with conventional technologies, EC presents distinct advantages, including reduced reaction times, elimination of chemical additives, diminished sludge production, and relatively uncomplicated equipment [33] Consequently, EC is applied to treat effluents from refineries [34,35], restaurants [36,37], food processing facilities [38,39], and slaughterhouses [39,40]. Illustratively, Aiyd et al. [41] achieved 99% oil content removal with an energy consumption of 4.342 kWh/kg OC and an electrode consumption of 0.29 g under optimized EC conditions for refinery wastewater.

Recent research endeavors prioritize augmenting the efficiency, selectivity, and cost-effectiveness of EC. Significant advancements encompass the development of novel electrode materials, such as doped metal oxides [42] or carbon-based electrodes [43]. Concurrently, precise optimization of operational parameters is emphasized. Employing experimental design methodologies (e.g., Box–Behnken design (BBD), central composite design (CCD)), Behera et al. [44] successfully reduced turbidity from 450 NTU to 56 NTU and total suspended solids (TSS) from 300 mg/L to 102 mg/L in oily wastewater, although economic viability necessitates further enhancement. Furthermore, integrating EC with complementary water treatment processes demonstrates considerable promise. Hybrid systems combining EC with ultrasound, membrane filtration, or photocatalysis exhibit notable synergistic effects, enabling more substantial reduction of residual oil content. Specifically, Nassar et al. [45] utilized ultrasound-assisted EC for restaurant wastewater, attaining enhanced removal efficiencies of 95.4% for oil/grease and 75.9% for COD. Their systematic investigation into parameters such as electrode spacing, electrolysis duration, and current density underscores the significant application potential of integrated EC processes for complex oily wastewater remediation.

### 3.2. Adsorption Method

Adsorption involves two distinct processes: the first refers to the adhesion of molecules to the surface of a solid due to chemical and electrostatic interactions, while the second, absorption, describes the assimilation of molecules into the internal structure of the adsorbent [46]. The adsorption method utilizes porous solids, known as adsorbents, to capture organic pollutants from oil-contaminated wastewater until the adsorbent reaches its maximum adsorption capacity or saturation point [47]. In this process, the molecular forces between the fluid and the adsorbent are pivotal, as they determine whether the liquid permeates the entire volume of the adsorbent or only adheres to its surface (i.e., adsorption) [48].

While the adsorption method is effective in removing oily substances, it faces several limitations, including susceptibility to saturation, high costs, and limited reusability. Current research on adsorption techniques for oil-contaminated wastewater largely focuses on developing novel adsorbent materials and modifying existing ones to improve their efficiency and capacity [49]. Traditional adsorbents, such as zeolites and activated carbon, are often hindered by high material costs, long adsorption times, and limited adsorption capacity. To address these issues, researchers have introduced cost-effective and highly efficient alternatives, such as foams, biomass, and metal–organic frameworks, which are well suited for the large-scale, continuous adsorption of organic or oily pollutants.

#### 3.2.1. Activated Carbon

Activated carbon is widely recognized as an effective adsorbent due to its well-developed porous structure, high specific surface area, and cost-effectiveness. However, the presence of numerous oxygen functional groups on its surface imparts significant hydrophilicity, which results in competitive adsorption between water molecules and pollutants. Hydrophilic materials have a strong affinity for water molecules, causing them to form a hydrated layer on the surface. This layer prevents oil droplets from directly contacting and adhering to the material, thereby reducing the efficiency of oil adsorption and separation. This competition reduces the adsorption efficiency of activated carbon for pollutants and accelerates its “aging” [50]. Additionally, the adsorption capacity of activated carbon is inherently limited, and the required reaction times can be prolonged. To address these challenges, researchers have begun exploring hydrophobic modifications of activated carbon to improve its adsorption efficiency.

Both thermal treatment and chemical grafting methods have been shown to effectively enhance the hydrophobic properties of inorganic materials. Gao et al. [51] reported that treating poplar wood with steam at 180 °C for 4 h resulted in a water contact angle of 125°. Similarly, Khurana et al. [52] modified zinc oxide nanoparticles with vinyl triethoxysilane (VTES), achieving a water contact angle greater than 150° following modification. Li et al. [53] applied hydrophobic modification to activated carbon using tetraethyl orthosilicate and trimethyl chlorosilane (TMCS), producing samples with a water contact angle of 143°.

Furthermore, given the limited adsorption capacity of activated carbon, there is growing interest in combining it with other processes to improve its performance. For instance, Li employed a combination of ozonation and activated carbon for treating refinery wastewater, achieving an oil removal rate of 48.2% after ozonation. When ozonation was followed by flocculation, the oil removal rate increased to 85.4%. Remarkably, the combination of ozone flotation and activated carbon adsorption resulted in an oil removal rate as high as 97.7% [54]. In the context of treating refinery wastewater through flotation, the addition of activated carbon—at doses ranging from 50 to 150 mg.g^−1^—led to increases in chemical oxygen demand (COD) and biochemical oxygen demand (BOD) removal rates from 16–64% and 27–70%, respectively, to 72–92.5% and 76–94%. Additionally, the magnetic modification of activated carbon used for treating oil-contaminated deep groundwater achieved an effluent oil concentration of less than 1 mg.g^−1^, with an oil removal efficiency of 96.7% [55].

#### 3.2.2. Sponges

The separation mechanism of sponges primarily involves physical adsorption. Due to their porous structure and high specific surface area, sponges can efficiently absorb the oil phase from oil–water mixtures, often holding oil several times their own weight. Additionally, sponges can be chemically treated to acquire oleophilic and hydrophobic properties, further improving their separation efficiency. After suitable cleaning, these sponges can be reused, which helps significantly lower processing costs.

(1) Polyurethane Sponge

Polyurethane (PU) sponge is a high-molecular weight thermosetting polymer formed through the esterification reaction between polyisocyanates and polyols. Due to its high porosity, excellent resilience, low density, high adsorption capacity, and ease of fabrication, PU sponge is the most widely used and extensively studied type of sponge. To meet specific practical application requirements, modification of the PU sponge is necessary. Post-modification, the wettability of the PU sponge is altered, while its ability to absorb oils and organic solvents remains intact, with the absorbed substances being recoverable by squeezing the sponge.

**Figure 2:**
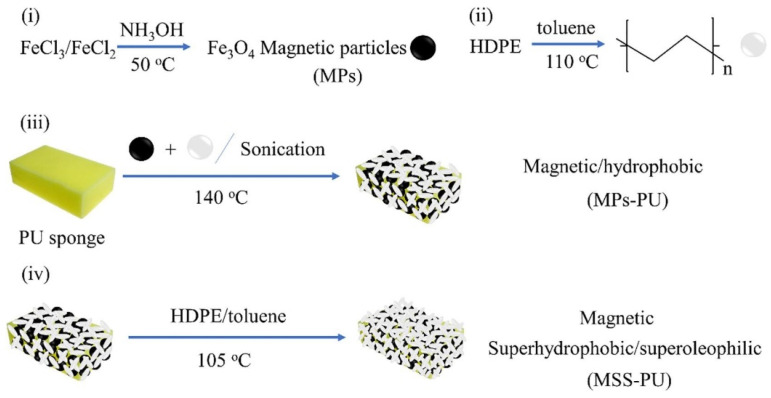
Schematic illustration of the preparation process for superhydrophobic/superoleophilic modified sponge (MSS-PU): (**i**) formation of magnetic particles by the co-precipitation reaction of Fe^2+^ and Fe^3+^ salts in alkaline medium; (**ii**) preparation of HDPE solution in toluene at 110 °C, (**iii**) immersion of the PU sponge into HDPE/MPs solution, followed by annealing at 140 °C for 1 h, (**iv**) further modification with HDPE/toluene solution and thermal annealing at 105 °C for 1 h. [57].

Numerous modification studies have demonstrated that PU sponge exhibits exceptional performance and great potential for treating surface-floating oils and separating emulsions. Sun et al. [56] coated the framework of a porous polyurethane sponge with a zinc oxide/epoxy resin solution, followed by a reaction with a mixture of stearic acid, acetic acid, and ethanol. This resulted in a superhydrophobic PU sponge with a water contact angle of 156 ± 3°. The modified sponge is capable of selectively separating mixtures of water and various oily solvents, with an absorption capacity for oily solvents ranging from 29 to 54 times its own weight. Yu et al. [57] immersed a commercial PU sponge in a high-density polyethylene (HDPE) solution containing magnetic (Fe_3_O_4_) particles, creating a superhydrophobic/superoleophilic modified sponge (MSS-PU), which demonstrated excellent oil–water separation ability, as shown in Figure 2. Parsaie et al. [58] prepared a superhydrophobic/superoleophilic PU sponge by applying a magnesium stearate coating and phenolic resin, achieving a water contact angle exceeding 175° and an absorption capacity of 19–381 g/g for various oils and organic solvents.

**Figure 3:**
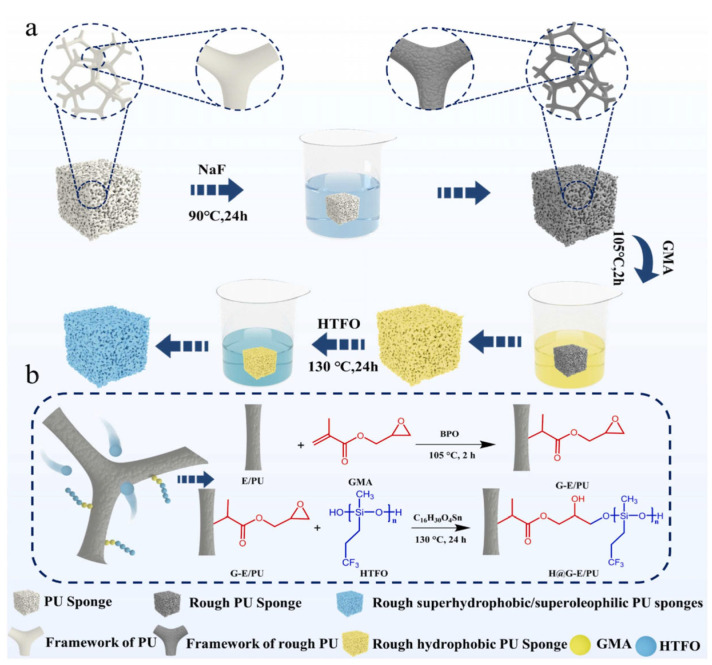
Fabrication schematic of superhydrophobic/superoleophilic RSS-PU sponge: (**a**) operation process (**b**) reaction mechanism [59].

However, these methods often face challenges such as the use of expensive reagents, complex preparation processes, and high toxicity. As a result, there is a clear need for the development of multifunctional, cost-effective, and environmentally friendly modified PU sponges. Xu et al. [59] explored a low-cost modified polyurethane sponge (RSS-PU), which was modified through sodium fluoride etching and end-capped with fluorosilicone oil. The modified sponge achieved a water contact angle of 151°, with separation efficiencies of 94.13% for mixtures without surfactants and 82.74% for emulsions containing surfactants, as shown in Figure 3. Furthermore, the flammability of PU sponges presents a risk in high-temperature environments. To address this, Zhang et al. [60] proposed a novel method to prepare superhydrophobic, flame-retardant, and magnetic polyurethane sponge (SFRM-PUS), which exhibited a contact angle of 156° and the ability to adsorb 42.6 times its weight in toluene. The physical barrier effect of reduced graphene oxide/iron oxide and the catalytic charring effect of phytic acid allowed the flame to be extinguished within just 4 s. These functional, low-cost superhydrophobic polyurethane sponges demonstrate significant potential for applications in oil spill cleanup and industrial wastewater treatment.

(2) Melamine Sponge (MS)

Melamine sponge (MS) is a three-dimensional porous material known for being lightweight, highly porous, mechanically robust, and thermally stable [61,62]. Similar to PU sponge, MS also exhibits amphiphilic properties, which require hydrophobic modification to improve its selective adsorption capacity and overall adsorption volume. Furthermore, its excellent resilience enables the recovered oil to be extracted by simply squeezing the sponge.

To attain superhydrophobicity, the melamine sponge framework is frequently modified with nanomaterials. Commonly utilized nanomaterials include titanium dioxide [63], graphene [64], nanodiamonds [65], silicon dioxide [66], carbon dots [67], etc., as illustrated in Table 1 below.

**Table 1:** Commonly used nanomaterials and their modification effects.

Nano-Sized Materials	Modification Method	Absorption Capacity (g/g)	WCA	Refs
Titanium dioxide	Spontaneous	77–211 times its weight	149° ± 1.5°	[63]
Graphene	Dip coating and chemical reduction	GO@MS: 105.3 times its weight rGO@MS: 72.3–136.5 times its weight	151°	[68]
Nanodiamonds	Interfacial assembling	26.65–55.64	155° ± 2°	[65]
Silicon dioxide	Dip coating and thermocuring	40–90 times its weight	162.6°	[69]
Carbon dots	Dip coating and thermocuring	—	—	[67]

In recent years, alongside nanomaterials, metal–organic frameworks (MOFs) have gained attention as an ideal choice for modifying oil adsorption materials, owing to their exceptional hydrophobicity, chemical stability, and high porosity. MOFs are effectively incorporated into sponge structures via in situ growth and surface functionalization, which significantly improves both selective adsorption capacity and stability.

The synergistic effect between MOFs and melamine sponge (MS) has proven highly effective in capturing and separating specific pollutants from complex mixtures. Taking advantage of this synergy, MOF materials, such as ZIF-8, Co-ZIF-L [65], MIL-53(Fe) [70], and MIL-Fe [71], are commonly used to modify MS. For instance, Lei et al. [72] developed a highly porous and hydrophobic composite MF-ZIF-8 sponge, which was integrated into the melamine framework through both non-covalent adsorption and covalent reactions. The addition of polydopamine, known for its strong adhesive properties, enabled effective immobilization of ZIF-8, resulting in enhanced catalytic efficiency.

Moreover, in recent years, a novel type of nanoporous carbon structure, derived from the thermal degradation of MOFs in inert gases, has garnered significant attention. Bauza et al. [73] coated commercial melamine-formaldehyde sponge surfaces with MOF-derived porous carbon, successfully creating highly hydrophobic and oleophilic sponges. These hybrid sponges, with their distinctive porous structures and exceptional properties (hydrophobic contact angle of 145° ± 6° and oleophilic contact angle of 0°), demonstrate excellent selectivity, high oil adsorption capacity for oil–water separation, and good recyclability.

The modification of melamine sponge by incorporating both low surface energy and high roughness offers an innovative approach in separation and adsorption technologies [74]. Whether applied individually or in combination, these two characteristics significantly enhance the sponge’s performance, clearly illustrating the synergistic effect between low surface energy and high roughness.

Despite the promising performance of MOF sponges in oil–water separation, challenges related to long-term stability and cost remain. Ensuring the sustained stability of MOF-modified sponges and addressing issues such as leaching or degradation are critical for their practical application [75]. As such, future research should focus on optimizing the synthesis process of MOF sponges to improve both their cost-effectiveness and environmental adaptability.

(3) Wood-Based Sponges

Wood-based sponges are lightweight, biodegradable, and highly compressible three-dimensional aerogels that have garnered significant attention in recent years as innovative adsorbents [76]. Their structure is primarily composed of cellulose, a readily available, low-cost, and environmentally friendly material, making them highly regarded in the field of sustainable development.

The structure of wood is inherently directional and hierarchical. Following chemical treatment to remove lignin and hemicellulose from the cell walls, a rigid cellulose framework remains, ensuring the material’s stability. As cellulose is the primary component of wood, it forms a cellulose aerogel that exhibits specific solvent adsorption capabilities and exceptionally high porosity. This unique property imparts wood-based sponges with excellent characteristics similar to those of traditional sponges, which is why they are referred to as wood-based sponges [77]. These sponges, derived from natural wood, can serve as substitutes for SiO_2_ and synthetic polymer sponges (such as polyurethane and melamine). Additionally, wood-based sponges maintain the biocompatibility of wood, offering a distinct advantage in environmentally friendly adsorption applications.

Wood-based sponges have demonstrated significant potential for absorbing organic solvents [78,79]. Due to their hydrophilic and oleophilic properties, they require specialized wettability modifications for applications such as oil–water separation and the absorption of oil or organic solvents. These modifications are typically achieved through chemical deposition methods. Once modified, wood-based sponges can not only filter sewage containing crude oil but also selectively absorb it. Liu et al. [80] developed superhydrophobic, recyclable, and regenerable functional wood sponges (SHWSs) from anisotropic natural balsa wood using a scalable, sustainable liquid phase deposition (LPD) silanization reaction. The resulting SHWSs exhibited excellent superhydrophobicity in hot water, alkaline, acidic, and saline environments, showing remarkable durability. They used SHWSs to construct a continuous oil–water separation device, achieving a flux of up to 72.8 L/h g and high separation efficiency. Wang et al. [81] prepared a wood-based sponge with 96.47% porosity from low-density balsam wood by treating it with a sodium hypochlorite solution at pH 4.0, 7% sodium hydroxide, and 7% sodium hypochlorite, followed by silanization. This sponge exhibited an oil and organic solvent adsorption capacity ranging from 2441 to 17,300 mg/g. Cai et al. [82] prepared a wood-based sponge by extracting lignin and hemicellulose from untreated poplar wood, followed by freeze-drying. To enhance the sponge’s mechanical compressive strength and hydrophobicity, they immersed it in a polyvinyl alcohol (PVA) solution and subsequently in a polydimethylsiloxane solution. This sponge demonstrated a water contact angle of 138°, an oil absorption capacity of 25 g/g, and exhibited both hydrophobic and oleophilic properties.

In contrast to the hydrophobic and oleophilic wood-based sponges mentioned earlier, Wu et al. [83] developed a hydrophilic wood-based sponge through catalytic oxidation. This process oxidized the hydroxyl groups on the surface of the wood sponge, converting them into carboxyl groups, resulting in a carboxylated wood sponge. The modified material achieved an impressive separation efficiency of 99.99% for crude oil–water emulsions and demonstrated a water absorption capacity of 15 g/g.

However, the oil absorption capacity of these sponges remains constrained by the material’s inherent structure and the preparation process. In practical applications, achieving optimal oil–water separation requires the use of a large quantity of wood-based sponges, which increases both costs and operational complexity. Additionally, during use, wood-based sponges are prone to physical wear and chemical corrosion, compromising their durability. Their soft and elastic nature makes them susceptible to deformation under external forces, which in turn impacts the effectiveness and accuracy of oil–water separation. For the large-scale production and application of wood-based sponges, future research must focus on further enhancing their preparation processes.

#### 3.2.3. Foam

In addition to sponges, other porous foam materials have also shown considerable potential for oil–water separation applications. The key advantage of foam materials is their ability to separate oil and water through mechanical compression after becoming saturated with absorbed substances, making them ideal for static, small-scale applications, such as in laboratories or for the preliminary treatment of small volumes of industrial wastewater. In contrast, sponge materials are better suited for applications that demand high elasticity and reusability.

Polyurethane foam (PUF) possesses a loose, porous structure with large internal cavities, creating optimal conditions for oil absorption [84,85,86]. However, traditional polyurethane foam materials exhibit poor hydrophobic properties, requiring hydrophobic treatment to effectively manage oil contamination.

Research has shown that modifying polyurethane foam can significantly enhance its adsorption capacity for oil–water separation, with the added benefit of improving its antibacterial properties. Navid Habibi et al. [87] developed a superhydrophobic layer on polyurethane foam by coating it with carbon black, hexagonal boron nitride, and acrylic resin, resulting in a water contact angle (WCA) of 159°. This modification enabled the foam to absorb up to 23 times its own weight, facilitating cyclic adsorption while preventing bacterial attachment, making it effective for oil–water separation in complex environments. Building upon this, Li et al. [88] functionalized polyurethane foam by attaching nano-copper oxide and n-hexadecylamine to its surface. The modified foam exhibited a WCA of 153°, although its separation efficiency decreased after 50 cycles of use. Alazab et al. [89] enhanced magnetic polyurethane foam by dip coating it with pre-treated cellulose-decafluorobiphenyl (MCF), which increased its absorption capacity to 9 to 32 times its own weight, along with an impressive separation efficiency (>97.68%). The modified foam also demonstrated excellent reusability (>50 cycles) in oil–water separation, maintaining high performance in challenging conditions, such as acidic (pH = 2) and alkaline (pH = 12) solutions, indicating its promising application potential.

Furthermore, improving the cyclic adsorption capacity of polyurethane foam facilitates multiple adsorption–desorption cycles. Zhang et al. [90] developed amino acid-doped antibacterial polyurethane foam (MWCNTs/PDA@AA-PUF) by coating a polydopamine/modified carbon nanotube composite onto amino acid-modified polyurethane foam. The modified AA-PUF exhibited an impressive absorption capacity, absorbing up to 23 times its own weight, while achieving separation efficiencies exceeding 97% for various oil–water and organic solvent–water mixtures. Moreover, it effectively prevented bacterial adhesion. This study demonstrated that AA-PUF, modified with PDA and MWCNTs, can serve as a highly efficient material for oil–water separation, with added antibacterial adhesion resistance.

While surface hydrophobic modification is an effective approach for improving foam material performance, it comes with high costs and, due to the weak bond between the hydrophobic agent and the material surface, the hydrophobic properties can degrade after repeated use. Furthermore, this modification only affects the surface, leaving the internal structure of the material unchanged, which limits its effectiveness in handling complex oil–water mixtures. As a result, smart switchable materials have gained increasing attention in recent research. Li et al. [91] developed a pH-responsive smart super-wetting iron foam material through electrodeposition and thiol modification, which demonstrated efficient oil–water separation under various pH conditions. After ten cycles, the separation efficiency remained above 98.5%. Table 2 presents a comparison of the effects of various modified materials on foam modification.

**Table 2:** Comparison of the effects of different modified materials on foam modification.

Modified Materials	Modification Method	Absorption Capacity (g/g)	WCA (°)	Refs
MCF	Dip Coating	9–32 times its weight	143	[89]
Nano CuO and HDA	Oxidative self-polymerization	45 times its weight	153	[88]
PDA and MWCNTs	Dip Coating	23 times its weight	153	[90]
HS(CH2)11CH3 and HS(CH2)10COOH	Electrodeposition and Thiol Modification	-	154.6 ± 2	[91]
CB(h-BN)@Fe3O4 and acrylic resin	Dip Coating	64–176	159	[87]

#### 3.2.4. Natural Biomass Adsorbents

Natural biomaterials offer distinct advantages over the materials previously mentioned. Derived predominantly from renewable resources, they are both environmentally friendly and biodegradable. Upon disposal, they decompose into harmless substances, such as carbon dioxide and water, without causing secondary environmental pollution. Additionally, bioadsorbents are often more cost-effective due to the abundant and readily accessible nature of their raw materials. As a result, the use of natural biomaterials for oil–water separation has attracted significant attention in recent years. These adsorbent materials are primarily obtained from biomass sources, including cellulose, chitosan, lignin, and others [92].

(1) Cellulose-Based Adsorbent Materials

Cellulose, the primary component of plant cell walls, is the most abundant natural organic material and a readily available renewable resource on Earth [93]. Naturally hydrophilic and highly porous, cellulose can be easily modified through simple chemical processes to acquire superhydrophobic or superoleophilic properties, making it highly effective for adsorbing organic pollutants like oils and cyclohexane. Cellulose-based adsorbent materials are generally classified into three categories: (a) cellulose-based aerogels, (b) cellulose-based smart responsive and adsorption-assisted materials, and (c) cellulose superhydrophobic sponges.

(a) Cellulose-Based Aerogels: Due to their ultralow density, highly branched three-dimensional nanostructures, and exceptional absorption capacity for various oils and organic solvents, cellulose-based aerogels exhibit significant potential in oil–water separation applications. A carbon aerogel derived from winter melon, prepared via hydrothermal treatment, freeze-drying, and subsequent pyrolysis, boasts a porosity exceeding 97.5% and a density of only 0.048 g/cm³, demonstrating excellent hydrophobicity and oleophilicity [94]. This aerogel can absorb 16 to 50 times its weight in oil and retains nearly 100% of its initial oil absorption capacity even after several recycling cycles. Similarly, carbon aerogels derived from durian shells and bamboo pulp also display exceptional oil absorption performance and reusability [95,96]. Additionally, nanocellulose-based aerogels, modified with surface treatment techniques such as sulfonation, can achieve underwater superoleophobicity, along with good cyclic stability and stable wettability. These properties make cellulose-based aerogels highly promising for the development of materials for oil–water separation and purification.
(b) Cellulose-Based Smart Responsive and Adsorption-Assisted Materials: The growing demand for advanced materials in various applications has led to increased interest in smart, responsive cellulose aerogels with tunable wettability, particularly for oil–water separation. Due to its highly porous network structure, cellulose not only enables control over the carbonization process for producing cellulose-derived materials but also facilitates the synthesis of cellulose-based aerogels, which can function as effective adsorbents. Li et al. [97] developed CO_2_-responsive cellulose nanofiber aerogels using surface-initiated atom transfer radical polymerization (ATRP) technology. The wettability of this aerogel can be modulated in response to environmental conditions. In the absence of CO_2_, the aerogel exhibits a water contact angle (WCA) of approximately 130°, making it hydrophobic. Upon CO_2_ exposure, the charged and extended PDMAEMA brushes form hydrogen bonds with water molecules, which reduces the effective pore size, enabling the aerogel to effectively separate water from oil–water mixtures. The aerogel’s porous structure and responsiveness contribute to its superior performance in oil–water separation. Moreover, its recyclability minimizes the impact of pollutants, significantly enhancing its reusability.

Meanwhile, Li et al. [98] developed an adsorption-assisted biomass-derived carbon aerogel by carbonizing sugarcane. This aerogel demonstrated significant hydrophobicity and oleophilicity without requiring chemical modification. Leveraging solar energy, its adsorption capacity increased from 31.9 g/g to 55.02 g/g, showcasing its exceptional photothermal conversion ability and enabling effective separation of high-viscosity oil–water mixtures. These findings highlight the promising potential of cellulose-based smart responsive and adsorption-assisted materials for oil–water separation. They not only offer advantages such as low cost, non-toxicity, and environmental friendliness but also exhibit excellent separation performance and recyclability.

(c) Cellulose Superhydrophobic Sponges: Due to their excellent mechanical properties, biodegradability, environmental friendliness, and low cost, cellulose superhydrophobic sponges have gained significant attention for oil–water separation applications in recent years. Meng et al. [99] developed a hydrophobic cellulose sponge through a simple preparation process, using cellulose (C_6_H_10_O_5_) that was etched with HCl and subsequently modified with octadecyltrichlorosilane (OTS) to enhance the sponge’s surface roughness and hydrophobicity. The sponge exhibited a water contact angle (WCA) of 153.5° and an oil contact angle (OCA) of 0°. It achieved a separation efficiency of up to 92% for various oil–water mixture systems. In another study, Qin et al. [100] coated commercial cellulose sponges with reduced graphene oxide (rGO) and polybenzoxazine (PBZ) to create nanocomposite cellulose sponges. These sponges displayed superhydrophobicity with a WCA of 155° and excellent mechanical stability over 100 compression cycles. They maintained a high oil–water separation flux (107,428 L m^−2^ h^−1^) at 0.10 bar and achieved a separation efficiency of 99.1% even after 10 cycles. Additionally, Li et al. [101] prepared smart responsive cellulose acetate sponges with switchable wettability under varying pH conditions, utilizing dynamic covalent amide bonds. These sponges exhibited a selective oil adsorption capacity of 40–80 times their weight and an 80% desorption ability after 10 separation cycles.

(2) Chitosan-Based Adsorbent Materials

Chitosan, a biopolymer derived from crustaceans such as crabs, shrimp, and crayfish, is increasingly used in oil–water separation due to its environmental friendliness and renewability. Yi et al. [102] prepared chitosan aerogels using directional freezing technology and applied superhydrophobic coatings via chemical vapor deposition (CVD). These aerogels demonstrated an impressive capacity to absorb up to 63 times their own weight in organic solvents and oils, showcasing excellent mechanical durability and reusability. Yin et al. [103] fabricated a superhydrophobic and magnetic chitosan-based aerogel using electrostatic interactions and dip coating methods, which effectively separated immiscible oil–water mixtures. Wang et al. [104] developed a salt-resistant superhydrophobic composite aerogel (SA/NSCS aerogel) that achieved a separation flux of 23,544 L m^−2^ h^−1^ and a separation efficiency of up to 99%. In another study, Liang et al. [105] prepared chitosan-based sponges through cross-linking methods, which selectively absorbed various oils and organic solvents, while exhibiting good elasticity and reusability. These studies underscore the broad application potential of chitosan-based adsorbent materials in water pollution control and oil spill cleanup.

(3) Lignin-Based Adsorbent Materials

Lignin is a natural polymeric material with varying content depending on the plant source. It is rich in chemical functional groups, including aromatic rings, phenolic hydroxyl groups, alcoholic hydroxyl groups, and carboxyl groups and has a complex three-dimensional network structure. These characteristics contribute to its broad potential for applications in oil–water separation [106]. However, due to its complex molecular structure and low chemical reactivity, directly utilizing lignin-containing agricultural and forestry waste for oil–water separation materials is challenging. Chemical modification, however, can significantly enhance the hydrophobicity and oil adsorption capacity of lignin-based materials. In addition to efficient oil–water separation, these materials offer the advantages of renewability, environmental friendliness, and low cost, providing a sustainable solution to oil–water pollution. Partow et al. [107] employed dimethyl sulfoxide as a solvent for lignin and grafted trimethylolpropane tris(3-mercaptopropionate) onto lignin molecules through a thiol-epoxy resin polymerization reaction, producing modified lignin aerogels. These aerogels demonstrated high mechanical resilience, chemical stability, and efficient adsorption of toxic organic solvents such as hexane, acetonitrile, and tetrahydrofuran. Unlike cellulose-based aerogels, lignin can be directly utilized to construct micro/nano structures. Tan et al. [108] successfully enhanced the porous structure and surface wettability of cellulose-based aerogels by incorporating lignin, which exhibited excellent underwater superoleophobicity and achieved an emulsion separation efficiency of up to 99%.

Aerogels created solely from lignin typically exhibit low adsorption capacity and limited reusability. To address these limitations, rigid lignin molecules can be combined with flexible polymer aerogels, resulting in lignin–polymer aerogels that offer high adsorption capacity, enhanced mechanical properties, and better recyclability. Sun et al. [109] developed a superhydrophobic porous material composed of lignin, n-hexadecyltrimethoxysilane, and melamine sponge through a simple impregnation method. This material not only adsorbs oil effectively but also facilitates continuous dynamic separation of oily wastewater, thereby eliminating time-consuming separation processes and reducing the need for adsorbents.

### 3.3. Filtration Methods

Filtration refers to the process in which a fluid is passed through a dense filtering medium. This method utilizes the inertia, sedimentation, and hydrodynamic effects of granular media, along with van der Waals forces, electrostatic interactions, and acid–base interactions, to effectively separate dispersed and emulsified oils from water. Typically, filtration is employed as a secondary or advanced treatment method in conjunction with other technologies to improve separation efficiency [110,111]. For instance, using filtration as a preliminary step can remove larger particles and reduce the load on subsequent adsorption or membrane processes, thus improving their effectiveness and lifespan. This integrated approach enables a more comprehensive treatment of water, ensuring a higher quality of separation and a more sustainable treatment process.

Deep-bed filtration is an advanced treatment method commonly employed for oily wastewater, particularly effective for treating low-concentration oily wastewater. This process consists of two primary stages: pollutant migration and adhesion [112]. The surface wettability of the filter media plays a crucial role in influencing oil removal performance. Filtration efficiency and head loss are the key indicators of deep-bed filtration performance. During filtration, filter media with stronger hydrophobic and oleophilic properties promote the adhesion of oil droplets, thereby enhancing oil removal efficiency but increasing head loss. Conversely, filter media with stronger hydrophilic and oleophobic properties allow water to more easily wet the surface, forming a water film that impedes oil adhesion, which reduces oil removal efficiency and also decreases head loss. Therefore, functional modification of the filter media to meet specific requirements is essential for optimizing performance.

In filtration systems, it is essential to consider the specific materials used. The choice of filtering media plays a crucial role in determining the efficiency and effectiveness of the filtration process. In the development of oil–water separation technologies, materials such as metal meshes, filter papers, foams, and membranes each offer distinct characteristics and potential applications, presenting various solutions for efficient oil–water separation [18]. Compared with adsorption methods, filtration is better suited for treating large oil droplets and suspended solids, offering the advantages of simplicity and cost-effectiveness. However, filtration media are susceptible to clogging by oils during oily wastewater treatment, which can lead to reduced efficiency. As a result, regular cleaning or replacement of the media is required. When selecting oil–water separation technologies, it is important to consider the specific properties of the wastewater (e.g., oil droplet size, oil–water ratio) and treatment costs in order to choose the most suitable method.

#### 3.3.1. Metal Meshes

Metal meshes are increasingly explored for the preparation of superhydrophobic materials due to their high mechanical stability, low cost, and versatility. Saengkaew et al. [113] developed superhydrophobic/superoleophilic surfaces on stainless steel meshes by coating them with natural rubber-encapsulated silica emulsions and modifying them with fluoroalkyl silanes for oil–water separation. Steel meshes, particularly those with superhydrophobic and superoleophilic properties, have gained popularity in oil–water separation applications owing to their mechanical strength, high separation efficiency, low cost, and easy availability. Recently, surface modification techniques for steel meshes have drawn significant attention. For instance, Chai et al. [114] coated steel meshes with hydrophobic and oleophilic silica particles to enhance oil–water separation. Zhang et al. [115] fabricated self-cleaning, underwater superoleophobic surfaces for oil–water separation by a layer-by-layer assembly of water glass and TiO_2_ nanoparticles on stainless steel meshes. Yang et al. [116] created superhydrophobic and superoleophilic steel meshes by spraying epoxy resin and palygorskite suspensions on stainless steel meshes, using carbon black and polypyrrole for modification. Dunderdale et al. [117] applied amphiphilic copolymers, such as poly(methyl methacrylate-co-ethylhexyl methacrylate), to functionalize stainless steel meshes for enhanced oil–water separation. Lee et al. [118] synthesized vertically aligned multi-walled carbon nanotubes on stainless steel meshes to improve oil–water separation efficiency.

Simple chemical vapor deposition methods can also be employed to modify metal meshes [119]. Deng et al. [120] prepared hydrophobic and oleophilic stainless steel meshes by coating them with a low-density polyethylene xylene solution. Liu et al. [121] treated stainless steel meshes by immersing them in copper chloride and hydrochloric acid solutions, followed by surface modification with stearic acid to create superhydrophobic and superoleophilic surfaces.

Chemical etching is a simple and cost-effective method for modifying metal meshes. Wang et al. [122] modified copper meshes by treating them with stearic acid after thermal oxidation, converting superhydrophilic copper meshes into superhydrophobic ones for oil–water separation. Varshney et al. [123] chemically etched steel meshes using a mixture of hydrochloric and nitric acids, followed by treatment with lauric acid, resulting in superhydrophobic and superoleophilic steel mesh surfaces (contact angle of 171 ± 4.5°). This surface design allows water to flow smoothly while enabling efficient oil permeation. The modified copper mesh demonstrated separation efficiencies exceeding 99% for petroleum ether–water and benzene–water mixtures. However, metal meshes also have certain limitations in practical applications, such as being heavy, prone to corrosion, and challenging to reuse.

Textiles are commonly used as selective oil–water separation filtration materials due to their low cost, lightweight nature, ease of processing, and corrosion resistance. Natural textiles, such as cotton, are renewable and biodegradable, making them ideal environmentally friendly base materials. Polylactic acid (PLA), a biopolymer derived from starch, is frequently used for modification due to its excellent biocompatibility and biodegradability, making it widely applicable in oil–water separation. Shen et al. [124] incorporated multiscale zeolite imidazole frameworks (ZIFs) onto PLA nonwoven fabrics and developed superhydrophobic materials through in situ growth and spray processes. Karthikeyan et al. [125] increased the surface roughness of PLA polymer meshes using a dip-dry method, converting their surfaces from hydrophilic to superhydrophobic. Ren et al. [126] proposed a cost-effective and environmentally friendly method for modifying PLA by immersing it in a suspension of titanium dioxide nanoparticles in xylene and trimethylpropyl silane. The modified fabric exhibited superhydrophobicity, and the PLA nonwoven fabric showed a 20% increase in organic adsorption capacity, with notable oil–water separation performance.

#### 3.3.2. Paper

Paper, primarily composed of cellulose, is an abundant material. Paper-based materials have gained considerable attention due to their lightweight nature, low cost, environmental sustainability, ease of fabrication, and versatile range of applications. These materials have been widely used to develop functional composites, with notable applications in electronics, sensing, and separation technologies. Among these, papers with tailored wettability properties offer distinct advantages in areas such as surface protection, long-term preservation, and oil–water separation.

Hydrophobic materials are commonly employed to modify nanoparticles, creating hierarchical structures with low surface energy and high roughness. Ogihara et al. [127] successfully developed highly hydrophobic and transparent superhydrophobic paper by applying a coating of hydrophobic silica nanoparticles. Similarly, Wang et al. [128] modified commercial filter paper using a mixture of hydrophobic silica nanoparticles and polystyrene solution in toluene, producing superhydrophobic and superoleophilic filter paper. This modified filter paper is effective for separating oils and low-surface tension liquids, such as ethanol, from water. However, the process involves hydrophobic modification of nanoparticles, indicating the need for further simplification of the procedure.

To overcome this challenge, Tang et al. [129] introduced a simpler and more widely applicable dip coating method. This technique incorporates silica nanoparticles and silane coupling agents onto the paper surface through a silanization process, resulting in highly hydrophobic filter paper capable of selectively filtering oil from mixtures. Experimental results demonstrated that the hydrophobic filter paper achieved separation efficiencies exceeding 98% for oil–water mixtures and emulsions, maintaining 98% efficiency even after 10 repeated cycles, indicating excellent stability.

Paper-based materials offer significant advantages, including large-scale producibility and low cost, which are crucial for practical applications. However, they also face challenges such as deformation and permeability. Furthermore, the regeneration and recycling of paper-based materials require further solutions to ensure their sustainability. Future research could focus on optimizing the formulation and processing of dip coating materials to improve the overall performance of paper-based materials.

### 3.4. Membrane Separation

Membranes are designed with specific pore sizes and operate based on principles of adsorption, sieving, and electrostatic interactions for separation. These processes are closely linked to the surface wettability, pore structure, and surface charge of the membrane materials. In recent years, membrane separation has emerged as a leading technology for oil–water separation, owing to its broad range of applications.

Membrane separation offers several advantages, such as low energy consumption, ease of operation, simple process flow, and low post-treatment costs. In comparison with traditional methods, membrane separation provides higher separation efficiency, which is largely dependent on the characteristics of the filter membrane. Recent advancements in membrane separation technology have enabled the large-scale treatment and purification of oily wastewater.

The preparation of membrane separation materials is primarily based on surface wettability, pore size, and surface charge of the filter membrane. Depending on the surface wettability, membranes can be categorized into superhydrophobic/superoleophilic membranes and superhydrophilic/superoleophobic membranes [130]. Superhydrophobic/Superoleophilic membranes are effective for filtering oil from oil–water mixtures and are typically used for oils with a higher density than water. In contrast, superhydrophilic/superoleophobic membranes are designed to filter water from oil–water mixtures and are most suitable for oils with a lower density than water.

Currently, common porous substrate materials include metal meshes, fibrous fabrics, ceramics, and synthetic polymer membranes. The surface wettability of these materials is controlled through methods such as spraying, dipping, solution phase deposition, chemical vapor deposition, and electrospinning [131]. These techniques create rough structures and modify surface energy, enabling selective filtration of oil or water.

#### 3.4.1. Metal-Based Filter Membranes

Metal-based filter membranes are oil–water separation materials fabricated with metal substrates, offering advantages such as high membrane flux, excellent mechanical strength, and scalability. Commonly used metal substrates in industry include stainless steel meshes, copper meshes, and titanium meshes. Copper meshes are highly reactive, which facilitates surface chemical reactions and modifications. Stainless steel meshes, being relatively inexpensive, exhibit good mechanical properties and greater ductility, allowing them to be woven into fine meshes with large mesh counts (e.g., over 1000 mesh), making them the most commonly used in oil–water separation. While titanium meshes have stable chemical and mechanical properties, their high cost and difficulty in weaving fine meshes limit their use in oil–water separation.

**Figure 4:**
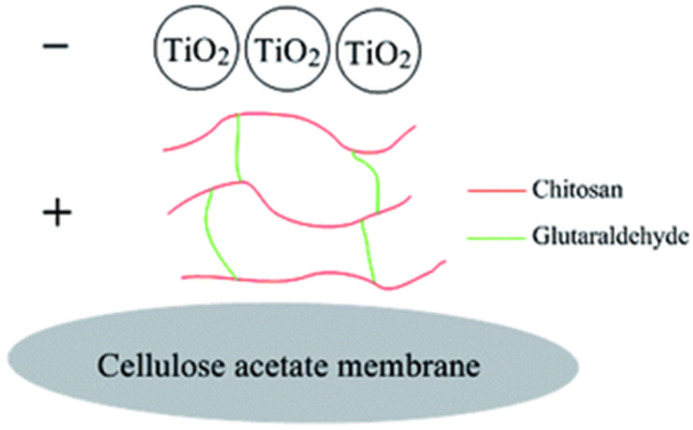
Schematic diagram of the preparation of chitosan-titanium dioxide composite membrane using vacuum-assisted filtration technology [134].

Qi et al. [132] developed a composite hydrogel membrane utilizing an inorganic CuO directional rod framework to support hydrophilic polyacrylic acid resin, achieving separation efficiencies exceeding 99.90% for mixtures of water with kerosene, hexadecane, soybean oil, and rapeseed oil and a water permeation flux of 5700 L m^−2^ h^−1^. In contrast, Avornyo et al. [133] observed that the use of Ag/CuO nanoparticles resulted in a water flux of only 303.63 L m^−2^ h^−1^, with the oil drainage efficiency dropping to 97.8%, highlighting the significant impact of different metal nanoparticles on membrane performance. Du et al. [134] fabricated a composite membrane by assembling chitosan and TiO_2_ on a cellulose acetate membrane using vacuum-assisted filtration technology, which achieved a flux of 6002.5 L m^−2^ h^−1^ for hexadecane emulsions in water, with a separation efficiency of over 97%, as shown in Figure 4. Studies indicate that TiO_2_-based membranes exhibit outstanding performance in oil–water separation, with separation efficiencies approaching 99%. Future research should focus on optimizing the preparation processes of metal-based filter membranes, particularly enhancing surface functionalization and performance. The introduction of various metal nanoparticles and innovative composite materials could further improve membrane selectivity and flux.

#### 3.4.2. Inorganic Non-Metallic Substrate Filter Membranes

Inorganic non-metallic composite membranes are highly valued for their high-temperature stability, corrosion resistance, wear resistance, thermal resistance, and oxidation resistance, all of which directly enhance their performance in oil–water separation. Specifically, high-temperature stability and thermal resistance ensure consistent performance under harsh conditions, while corrosion and oxidation resistance prolong the membrane’s lifespan in chemically aggressive environments. Additionally, wear resistance preserves structural integrity over extended use, rendering these membranes highly efficient and durable for oil–water separation.

Common inorganic non-metallic substrates used in oil–water separation include ceramics, carbon-based materials, and glass fiber fabrics. Ceramic membranes are particularly noted for their superior chemical, thermal, and mechanical stability, with common materials including silicon carbide, boron carbide, zeolite, alumina, zirconia, and titania. Among these, zirconia and alumina ceramic membranes are more widely applied. For example, Baig et al. [135] developed a functionalized carbide-derived carbon composite ceramic membrane through cross-linking, achieving a maximum flux of 76.05 L m^−2^ h^−1^ and oil separation efficiency exceeding 99%. While ceramic membranes offer excellent high temperature and corrosion resistance and allow for controllable pore sizes to meet diverse separation needs, they remain relatively expensive and brittle. Thus, further process improvements are required to enhance both their performance and cost-effectiveness.

Common carbon materials include carbon nanotubes (CNTs) and graphene oxide (GO). These carbon materials are not only modifiable on their own but can also form composites with inorganic particles and organic molecules to improve performance. Naseeb et al. [136] developed PAN-GO-SiO_2_ hybrid membranes using electrospinning technology. The membrane’s hierarchical structure, combined with the oxygen-containing groups on SiO_2_ and GO, enhanced its hydrophilicity, achieving a separation efficiency of up to 99% for oil–water emulsions, making it a promising area of research. However, the high cost and complex, time-consuming manufacturing processes associated with carbon nanomaterials limit their broader development and application.

#### 3.4.3. Polymer Filter Membranes

Polymers consist of repeating units known as monomers and can be classified into synthetic and natural types based on their origin. Synthetic polymers, which are chemically synthesized and not naturally occurring, serve as the foundation for synthetic polymer filter membranes through various modifications. These membranes provide advantages such as corrosion resistance and high tensile strength. Recently, materials like polyvinylidene fluoride (PVDF), polypropylene (PP), polyacrylonitrile (PAN), nylon, and their composites have become widely utilized for oil–water separation membranes. However, these synthetic polymers are non-biodegradable and may pose environmental risks. In contrast, nanofiber membranes offer superior anti-fouling properties and enhanced environmental performance.

Electrospinning is widely recognized as a flexible, effective, cost-efficient, and industrially feasible technique for membrane fabrication [137,138]. Since the late 1990s, the application of electrospinning technology to create nanofiber adsorption and filtration membranes has attracted increasing attention in the field of oil–water separation [12,139,140]. Electrospun nanofibers are characterized by their high surface area, highly interconnected porous structures, and nanoscale pore sizes [141]. The diameter of electrospun fibers can be controlled within a range of a few micrometers to several nanometers, making them highly effective for separating emulsified oil mixtures. Lin et al. [142] successfully controlled the micro/nano structure of electrospun polystyrene (PS) fibers by adjusting solution composition, concentration, and polymer molecular weight, significantly enhancing the oil absorption capacity of the porous PS fibers. Their adsorption capacities for engine oil and sunflower oil were 84.41 and 79.62 g g^−1^, respectively, three times higher than that of commercial polypropylene (PP) nonwoven fabric. Furthermore, copolymerization of styrene with other monomers is an effective method to enhance the performance of electrospun PS fiber membranes. Ning et al. [143] synthesized several polymers through the suspension polymerization of styrene and butyl acrylate, electrospinning these materials into fiber membranes. These membranes demonstrated excellent oil–water separation performance, with a contact angle of 155° and a maximum oil removal efficiency of 97.3%, maintaining 74.2% efficiency even after seven cycles. The membrane’s superior oil absorption capabilities contribute to clearer oil–water emulsions.

High-efficiency nanofiber membranes have gained significant attention due to their large surface area-to-volume ratio and recyclability [144]. Yang et al. [145] successfully prepared polystyrene (PS) nanofiber membranes functionalized with polydopamine by immersing PS pads in a dopamine alkaline buffer for 24 h. Oil–water separation was achieved through Michael addition reactions between polydopamine and undecanethiol (UT) or undecan mercaptodecanoic acid. The long alkyl chains of UT on the membrane surface enhanced the hydrophobicity, allowing oil to pass through while blocking water. In addition, Tang et al. [146] introduced an innovative approach to fabricate superhydrophobic and superoleophilic nanofiber membranes for gravity-driven separation of oil–water microemulsions. F-PBZ, prepared in situ on the surface of silica nanofibers, exhibited high flux (892 L m^−2^ h^−1^), excellent thermal stability, and durability, demonstrating the practicality of the composite membrane for emulsified oil–water separation.

**Figure 5:**
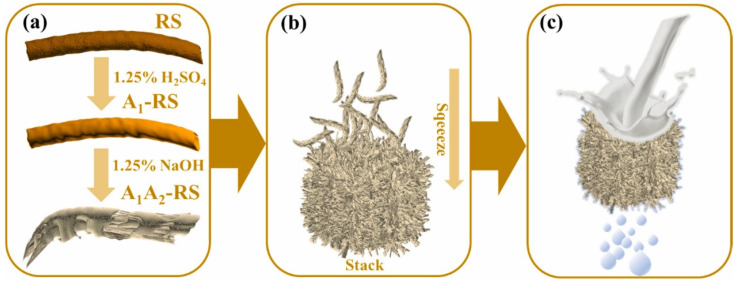
(**a**) Schematic diagram of the synthesis process of A1A2-RS using a simple acid–base treatment; (**b**) schematic diagram of the process for extrusion-assembled layers; (**c**) conceptual diagram of oil–water emulsion separation [147].

Natural polymers, including cellulose, cotton, hemp, and resins, are produced by living organisms in nature. Filter membranes produced from modified natural polymers offer benefits such as biodegradability, environmental sustainability, wide availability, and low cost. In contrast to synthetic polymer membranes, which are non-biodegradable and can enter the human body through the food chain, posing potential health risks, natural polymers present greater research value in terms of both environmental impact and human health. Feng et al. [147] developed a separation layer for oil-in-water emulsion filtration using a straightforward acid–base treatment and micro-squeeze process on straw, as depicted in Figure 5. The acid–base treated straw (A1A2-RS) displayed distinct fibrous structures and abundant protrusions, demonstrating excellent adsorption capacity for various cetyltrimethylammonium bromide (CTAB)-stabilized oil-in-water emulsions, with a membrane flux exceeding 500 L m^−2^ h^−1^ and separation efficiency greater than 97.5%. Zhang et al. [148] fabricated a wood-based composite membrane through a simple dip coating process, achieving over 99.3% efficiency in separating oil–water emulsions with a membrane flux of 227 L m^−2^ h^−1^, maintaining exceptional performance and stability even in complex environments, such as acids, bases, seawater, and high temperatures.

Cotton fabric filter membranes are derived from cotton fabric and modified to enhance specific properties. These membranes offer advantages such as lightweight construction and corrosion resistance. As a natural textile material, cotton is widely available and possesses environmentally friendly attributes, including biodegradability and recyclability. Hu et al. [149] developed a simple and effective method that combines surface micro-dissolution using NaOH/urea with in situ water vapor generation to grow and fix Mg(OH)_2_ onto the cotton fabric surface, resulting in a super-wetting cotton fabric (Mg(OH)_2_@CF). This material achieved a separation efficiency of 99.5% for oily wastewater under gravity-driven conditions, with a membrane flux ranging from 3027 to 4137 L m^−2^ h^−1^. It also achieved a separation efficiency of 99.5% for emulsified oils, with a membrane flux of 968 L m^−2^ h^−1^.

Ou et al. [150] synthesized a composite material, CC-ZIF-PIL, by grafting ZIF-8 nanoparticles and polyelectrolytes (PILs) onto commercial cotton fabric (CC) through in situ grafting. This composite effectively separated oil-in-water and water-in-oil emulsions, with organic solvents serving as the oil phase. The high porosity of ZIF-8 facilitated the adsorption of oil from oily wastewater and provided channels for its transport, thereby enhancing both the separation efficiency and membrane flux of CC-ZIF-PIL.

Compared with the membrane materials mentioned earlier, cellulose-based membranes offer distinct advantages, including lighter weight, greater flexibility, lower cost, easier processing, and enhanced renewability. Additionally, they are environmentally friendly and biodegradable. These characteristics make cellulose-based materials highly promising as alternatives to metal and synthetic polymer substrates in the field of oil–water separation.

**Figure 6:**
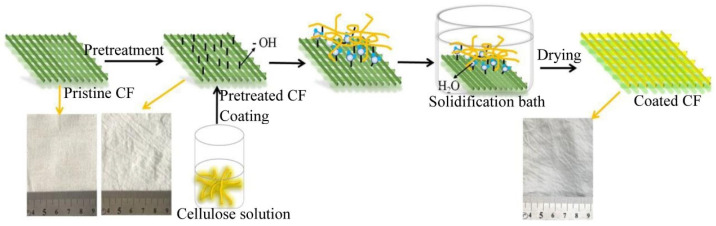
Schematic diagram of the preparation process of coated cotton fabric (Coated CF) [151].

Cellulose, the most abundant natural polymer, contains numerous hydrogen bonds within its molecular structure, which makes it highly hydrophilic and capable of being dissolved and regenerated. The regenerated material forms micro- and nano-sized pores. Commercial cellulose is primarily extracted from wood or cotton, although it can also be sourced from agricultural waste. In 2013, cellulose-based polymers constituted 61.8% of the total biopolymer production. Life cycle assessments suggest that integrating biopolymer membranes with eco-friendly processes can help reduce environmental impacts. Zhang et al. [151] developed a filter membrane by dissolving and regenerating cellulose onto commercial cotton fabric (CF), resulting in a composite structure, as depicted in Figure 6. The dissolution of cellulose in a NaOH/urea solution, followed by regeneration in a salt solution, produced strong adhesion to the cotton fabric, creating a “sandwich” structure. This modification significantly improved the strength, thermal stability, hydrophilicity, and underwater oleophobicity of the cotton fabric, allowing it to efficiently separate highly emulsified oil–water mixtures, with organic solvents serving as the oil phase.

The chemical structure of microbial cellulose closely resembles that of plant cellulose, as it is produced by aerobic bacteria in the form of a hydrated membrane outside the cell [110,152,153,154]. Bacterial cellulose (BC) is a natural biopolymer with a broad range of applications. It is produced by various microorganisms, either individually or in combination. Research has demonstrated that BC has the potential to replace traditional methods of plant cellulose production, positioning it as a promising biopolymer [155,156,157]. Donini et al. [158] emphasized that BC possesses excellent mechanical properties, high crystallinity, substantial water retention capacity, a high degree of polymerization, and good biodegradability. A notable advantage of BC membranes is their washability; saturated BC membranes can be removed from filtration systems, washed, and reused without compromising filtration efficiency [159]. Galdino et al. [160] confirmed that BC membranes effectively remove oil from wastewater and can be washed and reused more than 20 times without losing their structural integrity or filtration performance.

However, the low porosity of the membrane and the size of oil molecules pose a challenge, as this leads to membrane saturation. Although various attempts have been made to modify the structure of BC through different production or processing methods, effective solutions for membrane protection and optimization of filtration rates remain lacking. There is an urgent need for researchers to identify polymer membranes with anti-fouling and/or antibacterial properties to enhance industrial wastewater purification.

## 4. Conclusions and Outlook

Recent advancements in oil–water separation technologies have been driven by the rapid pace of industrialization and growing environmental awareness. This paper reviews traditional oil–water separation techniques, such as gravity separation, centrifugal separation, and flotation, alongside emerging methods like adsorption, filtration, and membrane separation. Special attention is given to innovative applications utilizing superhydrophobic/superoleophilic materials and multifunctional composites. The advantages and disadvantages of the methods and materials described above are as illustrated in the Table 3 below. The development of these novel materials and technologies not only improves the efficiency of oil–water separation but also contributes to reducing environmental impacts and processing costs.

However, contemporary research in oil–water separation continues to encounter several significant challenges. First, there is a need to significantly improve the separation efficiency for complex oil–water systems, such as emulsified oils and wastewater containing surfactants, to enable their practical application. Second, the durability and stability of separation materials under extreme environmental conditions, including high temperatures, strong acidity or alkalinity, and high salinity, remain insufficient. Moreover, the high production costs of certain high-performance materials hinder their widespread use in large-scale applications. Finally, our understanding of the microscopic mechanisms underlying the interactions between materials and contaminants, particularly at the molecular level, remains limited, which complicates material design and performance optimization.

To address these challenges, future research in oil–water separation technology should focus on several key areas: (1) The development of intelligent, responsive materials capable of adjusting their surface properties in response to environmental factors such as pH, temperature, and salinity, achieved through molecular design and surface modification, to improve separation efficiency and adaptability. (2) Enhancing material durability by exploring more stable modification techniques to boost corrosion resistance and mechanical stability in complex environments, thereby extending the lifespan of the materials. (3) Optimizing manufacturing processes to lower the production costs of high-performance materials while streamlining workflows and improving material recyclability and environmental sustainability. (4) Conducting comprehensive studies of the microscopic mechanisms governing material–contaminant interactions through both experimental and simulation approaches, providing a theoretical foundation for the design of advanced, high-performance separation materials.

Furthermore, the integration of multiple separation techniques to create efficient and consolidated oil–water separation systems will become an important research focus. For example, combining membrane separation technology with adsorption or filtration methods can improve separation efficiency while reducing the risk of membrane fouling. At the same time, advancing the use of green and biodegradable materials to reduce dependence on conventional materials is essential for developing sustainable oil–water separation technologies.

In conclusion, the progress of oil–water separation technologies requires a collaborative approach that combines material innovation, process optimization, and theoretical research. By fostering interdisciplinary cooperation and integrating various technologies, the goal is to develop more efficient, environmentally sustainable, and cost-effective solutions to tackle the growing environmental challenges.

## Figures and Tables

**Table 3:** The advantages and disadvantages of the methods and materials.

Technology	Advantages	Disadvantages
Gravity Separation	Simple and low-cost, suitable for large-scale wastewater treatment Effective for separating oil droplets larger than 10 μm	Low separation efficiency for small oil droplets Ineffective for treating stable emulsions
Centrifugal Separation	High separation efficiency for fine oil droplets Fast processing speed	Complex equipment and difficult maintenance High energy consumption and cost
Flotation	Can treat fine oil droplets and emulsified oil	Requires addition of chemical agents, potential secondary pollution
Electrocoagulation	High separation efficiency and simultaneous removal of multiple pollutants Minimal chemical additives required	High energy consumption and electrode material costs Electrodes prone to scaling and require regular replacement
Adsorption	Diverse adsorbents with strong selectivity High adsorption efficiency and recyclability	Limited treatment capacity for high-concentration wastewater
Filtration	Simple operation, suitable for large-scale treatment Can be combined with other technologies to enhance separation efficiency	Prone to filter media clogging, requiring regular replacement Limited effectiveness for emulsified oil treatment
Membrane Separation	High separation efficiency and low energy consumption Simple operation, suitable for various oil–water systems	High membrane material costs and prone to fouling Regular membrane cleaning or replacement required
Superhydrophobic/ Superoleophilic materials	High separation efficiency and effective oil–water separation Diverse materials with strong designability	Some materials have poor stability and durability Limited adaptability to complex wastewater
Stimuli-Responsive Materials	Can automatically adjust separation performance based on environmental changes	Long-term stability requires further verification

## Data Availability

No new data were created or analyzed in this study.
